# ED-B fibronectin expression is a marker of epithelial-mesenchymal transition in translational oncology

**DOI:** 10.18632/oncotarget.13615

**Published:** 2016-11-25

**Authors:** Iacopo Petrini, Serena Barachini, Vittoria Carnicelli, Sara Galimberti, Letizia Modeo, Roberto Boni, Martina Sollini, Paola Anna Erba

**Affiliations:** ^1^ General Pathology, Department of Translational Research and New Technology in Medicine, University of Pisa, Pisa, Italy; ^2^ Laboratory of Hematology, Department of Clinical and Experimental Medicine, University of Pisa, Pisa, Italy; ^3^ Biochemistry, Department of Translational Research and New Technology in Medicine, University of Pisa, Pisa, Italy; ^4^ Nuclear Medicine, Department of Translational Research and New Technology in Medicine, University of Pisa, Pisa, Italy; ^5^ Department of Biomedical Sciences, Humanitas University, Rozzano, Milan, Italy

**Keywords:** fibronectin, endothelial-to-mesenchymal transition, ED-B, prostate cancer, TGF-β

## Abstract

Fibronectin is a component of the extracellular matrix that links collagen fibers to integrins on the cell's surface. The splicing isoforms, containing the ED-B domain, are not expressed in adult tissues but only in tumor stroma or during embryonic development. Fibroblasts and endothelial cells express ED-B fibronectin during angiogenesis. Also cancer cells can synthetize ED-B fibronectin, but its function in tumor growth needs to be further elucidated.

We evaluated the expression of ED-B fibronectin in prostate cancer cell lines: PC3 and DU145. Using TGF-β, we induced epithelial to mesenchymal transition in culture and observed an increase of ED-B fibronectin expression. Thereafter, we evaluated the expression of ED-B fibronectin in multipotent mesangiogenic progenitor cells, and in mesenchymal stromal cells. The expression of ED-B fibronectin was much higher in mesenchymal than prostate cancer cells even after the epithelial to mesenchymal transition.

Epithelial to mesenchymal transition is a key step for tumor progression contributing to the metastatic spread. Therefore, circulating cancer cells could seed into the metastatic niche taking advantage from the ED-B fibronectin that secrete their own.

## INTRODUCTION

Fibronectin is an essential component of the extracellular matrix that is necessary for the cellular adhesion to the stroma. Indeed, it creates a bridge between the molecules of the cell surface (integrins) and the collagen fibers [1]. Fibronectin, through the binding with integrins, activates intracellular pathways leading to cell adhesion, growth and differentiation [2]. Moreover, fibronectin can induce cytoskeleton modifications, allowing cell migration, since integrins are anchored to the actin fibers through the molecules talin and vinculin [2]. Therefore, fibronectin is necessary for embryogenesis and wound healing. Fibronectin is a glycoprotein that contains 12 fibronectin type-I repeats, 2 fibronectin type-II repeats, 17 fibronectin type-III repeats and a non-homologous variable connecting segment: the IIICS. Two of the type III repeats can be alternatively spliced (ED-A and ED-B) [1]. There are at least 20 known different isoforms originating from a single pre-mRNA. The function of these isoforms remains obscure in most cases. ED-A and ED-B sequences are highly conserved among vertebrates [3] and these isoforms are abundantly expressed during embryogenesis supporting their role in vascular development, cell migration, and differentiation [4, 5]. In adults, ED-A and ED-B fibronectin expression is induced in specific conditions such as tissue repair, fibrosis, angiogenesis, and cell migration [4, 6]. Indeed, ED-B fibronectin has a restricted pattern of expression: it is almost undetectable in normal adult tissues and in mature blood vessels, but is abundant in regenerating tissues and around newly formed blood vessels [7, 8]. Mice lacking ED-A or ED-B domain grow-up without obvious defects [9]. Whereas, the simultaneous knock-out of both ED-A and ED-B exons determines embryonic lethality, with an incomplete penetrance, for the presence of multiple cardiovascular defects [9].

For their role in tissue remodeling, angiogenesis and migration, ED-A and ED-B isoforms could play a relevant role in cancer growth. Indeed, during the formation of metastasis, cancer cells have to seed in the metastatic niche through the binding of their membrane integrins with the fibronectin present in the extracellular matrix [10].

However, the exact role of ED-A and ED-B fibronectin in cancer remains largely obscure. ED-B fibronectin is expressed in tumor tissue [11], in particular in breast carcinoma [12], brain tumors [13], lymphoma [14] and prostate cancer [15]. High ED-B fibronectin expression was found around newly formed blood vessels and therefore could be a candidate target to treat tumor angiogenesis.

Accumulating evidences have shown that the Epithelial-Mesenchymal Transition (EMT) is necessary for cancer progression; especially for the metastatic spread [16–20]. During EMT, cells enhance the expression of mesenchymal markers together with the repression of epithelial molecules. Since fibronectin is a mesenchymal marker, we evaluated the expression of ED-B fibronectin during EMT in two prostate cancer cell lines.

## RESULTS

### TGF-β induces EMT in prostate cancer cell lines

**Figure 1:**
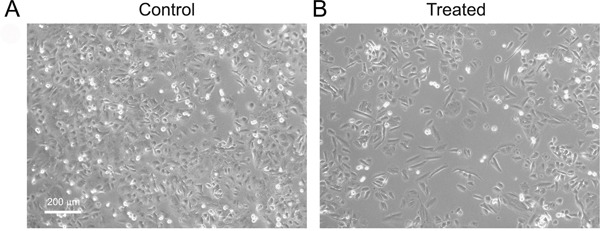
Morphology of Du145 cells treated with or without TGF-β After 2 weeks of treatment cells showed an elongated mesenchymal-like appearance. Pictures were taken by phase contrast microscopy.

EMT was induced, using TGF-β, in two cell lines of prostate cancer: PC3 and Du145. After 2 weeks of TGF-β treatment, cells with an epithelial-like morphology acquired a more elongated shape resembling that of fibroblast in culture: a typical characteristic of mesenchymal-cells (Figure 1).

**Figure 2:**
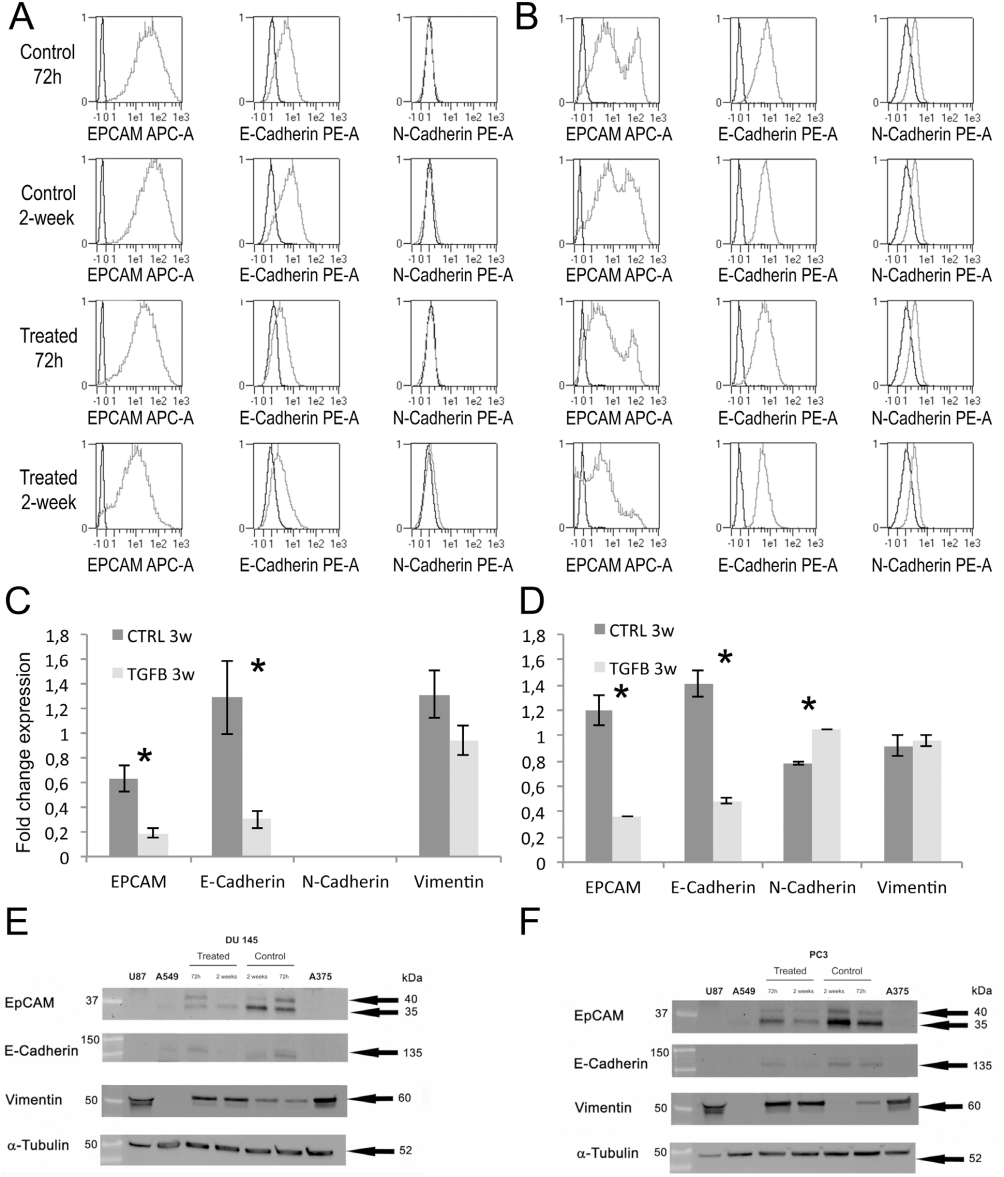
Analysis of EMT markers: cytofluorimetric analysis of Du145 A. and PC3 B. Gene expression of EMT markers after 2 weeks of treatment with or without TGF-ß in DU145 C. and PC3 D Western blot analysis of EMT markers in Du145 **E**. and PC3 **F**. cells after TGF-β treatment for 72h and 2 weeks. Protein lysates of U87 (human glioblastoma cell line), A549 (human lung adenocarcinoma cell line) and A375 (human melanoma cell line) were used as positive or negative controls.

Using flow cytometry, we observed a reduction in the expression of the epithelial markers EPCAM and E-Cadherin in PC3 and Du145 cells after treatment with TGF-β for either 72 hours or 2 weeks.Du145 cells expressing E-Cadherin were 30.6% (standard deviation (SD)±3.4) and 78.8%(SD±1.7) after 2 weeks of culture with or without TGF-β, respectively (Figure 2A). A milder effect was observed for Du145 cells expressing EPCAM that were 86.2%(SD±2.3) and 99.9%(SD±0.5) after 2 weeks of culture with or without TGF-β, respectively (Figure 2A). In line with previous reports [21, 22], there were not differences in the percentage of N-Cadherin expressing cells with or without TGF-β treatment (after 72h and 2-week incubation) (Figure 2A). PC3 cells expressing EPCAM were 40.8% (SD±2.6) and 90.4%(SD±1.2) after 2 weeks of culture with or without TGF-β, respectively (Figure 2B). A milder effect was observed for PC3 expressing E-Cadherin that were 97.0%(SD±3.7) and 95.6%(SD±3.4) after 2 weeks of culture with or without TGF-β, respectively (Figure 2A). There were not differences in the percentage of PC3 cells expressing N-Cadherin with or without TGF-β treatment after 72h and 2-week (Figure 2B).

Gene expression was evaluated by RT-PCR after 2-week of TGF-β treatment. In Du145 cells, TGF-β significantly reduced the expression of EPCAM (−70%; p=0.010) and E-Cadherin (−77%; p=0.023) compared to controls (Figure 2C). DU145 did not express N-Cadherin either with or without TGF-β treatment. TGF-β did not induce a significant difference in vimentin expression in DU145 (−28%; p=0.57). In PC3 cells, TGF-β significantly reduced the expression of EPCAM (−70%; p=0.007) and E-Cadherin (−66%; p=0.003) compared to controls (Figure 2D). TGF-β induced a significant increase of N-Cadherin expression in PC3 cells (+35%; p<0.001) but not significant differences in vimentin expression (+4%; p=0.522).

In Du145 and PC3, western blots demonstrated a decrease of EPCAM and E-Cadherin expression and an increase of Vimentin both after 72h and 2-weeks (Figure 2E, 2F). Western blots definitely demonstrated EMT showing the expected EPCAM and E-cadherin signal reduction alongside with vimentin increase.

### ED-B fibronectin expression in EMT and mesenchymal cells

**Figure 3:**
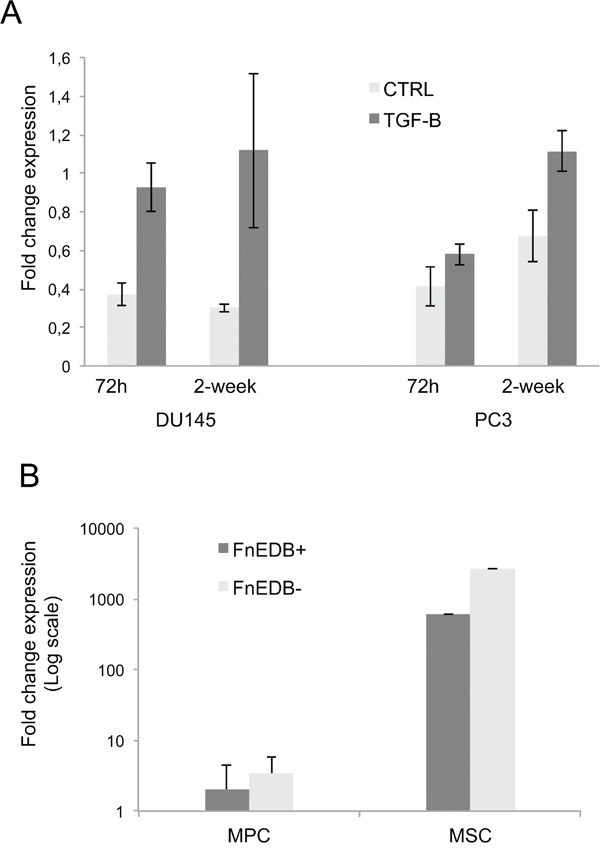
Fibronectin ED-B mRNA expression in Du145 and PC3 cell lines after 72h and 2 weeks **A**. Fibronectin ED-B expression in MPC, P2-MSC and Du145 treated for 2 weeks **B**.

TGF-β induced the expression of ED-B fibronectin. In DU145 cells, ED-B fibronectin expression was increased by 60% (p=0.007) after 72 h and by 73% (p=0.071) after 2 weeks of TGF- β treatment. In PC3 cells, ED-B fibronectin expression was increased by 29% (p=0.087) after 72h and significantly by 40% (p=0.012) after 2 weeks of TGF-β treatment (Figure 3A). Similarly there was an increase of total-Fibronectin with TGF-β (72h and 3w) in DU145 and in PC3 after 72h of treatment (data not shown).

The ED-B fibronectin expression in DU145 was much lower than in MSCs and MPCs (Figure 3B). Even if the EMT was induced, the transformed PC3 and Du145 did not show a prototypical mesenchymal phenotype such as MSC (EPCAM^neg^CD105^+^CD90^+^STRO-1^+^) or MPC (EPCAM^neg^CD105^neg^CD90^neg^CD31^+^).

## DISCUSSION

ED-B fibronectin expression increased during EMT. However, ED-B fibronectin expression was much higher in mesenchymal cells and their progenitor MPC compared to that of tumor cell lines undergoing EMT under the induction of TGF- β.

Interestingly, we observed the expression of ED-B fibronectin in prostate cancer cells. Fibronectin is a mesenchymal marker and increases in TGF-β–induced EMT [20, 23]. ED-B fibronectin was up regulated during EMT in Du145 and PC3 cells upon TGF-β induction. This is relevant for cancer cells’ migration and for their metastatic progression. Indeed, tumor cells can synthetize the fibronectin isoforms expressed during tissue remodelling and neo angiogenesis and could become independent from the expression of fibroblasts and mesenchymal cells. These two sources of ED-B fibronectin can contribute to create a permissive soil for the invasion, attachment and growth of metastases.

Physiologically, ED-B fibronectin is synthesized, secreted, and deposited into the extracellular matrix by numerous cell types such as endothelial cells of newly formed blood vessels and myofibroblasts [14, 24, 25]. Cells transformed *in vitro* using SV40 start to express ED-B fibronectin [26]. Moreover, fibronectin and ED-B mRNA transcripts are expressed in cancer cell lines including colorectal and breast carcinoma, among others [24]. The protein can not be described on the surface of these cells *in vitro*, probably because it is rapidly secreted. Indeed, ED-B fibronectin, secreted by cancer cells, accumulates abundantly into mouse xenograft according to the BC1 antibody, which is specific for human but not mouse fibronectin [24]. The FDC6 antibody recognizes an O-glycosylated epitope on a threonine, inside the IIICS domain, of a fibronectin isoform expressed in cancer or in fetal cells/tissues, but not in normal adult cells/tissues [27]. Lima et *al*. have observed the up-regulation of this fibronectin isoform during EMT in prostate and lung adenocarcinoma cell lines [27, 28]. Moreover, authors demonstrated that this O-glycosylated fibronectin isoform promotes the EMT processes [27].

ED-B fibronectin could represent an ideal target for cancer therapy because it is overexpressed in tumor but not in normal adult tissues. ED-B fibronectin has a different expression in different tumors in terms of intensity and distribution of the staining [29]. Certain cancers express high level of ED-B fibronectin (i.e. lung cancer, melanoma, colon cancer) whereas others seem to increase the ED-B isoform while they acquire a more aggressive phenotype (i.e. lymphoma, prostate cancer, thymoma/thymic carcinoma and thyroid cancer). Our group has already demonstrated ED-B fibronectin expression in lymphomas and leukemia [14]. ED-B fibronectin expression was higher in the wall of blood vessels of high-grade compared to low-grade non-Hodgkin lymphomas. Similarly, ED-B fibronectin was expressed in perivascular spaces of bone marrow samples with lymphoma or leukemia infiltration, whereas perivascular spaces of normal bone marrow were almost negative [14]. Low-grade prostate cancers presented a lower ED-B fibronectin expression than undifferentiated ones, indicating a correlation between the grade of differentiation and ED-B fibronectin expression. Therefore, we have chosen for these experiments PC3 and DU145: two cell lines derived from metastatic tumors that have lost their androgen dependency typical of the more differentiated prostate cancers. Anti-ED-B radiolabeled immunoconjugates have demonstrated *in vivo* the presence of ED-B fibronectin in tumor stroma of lymphomas and carcinomas including lung and prostate [14]. Therefore, the use of anti-ED-B radiolabeled immune-conjugates represents a promising approach to target cancer progression. The treatment of advanced tumors with ^131^I-L19 SIP direct against ED-B fibronectin is associated with thrombocytopenia of grade 3-4 [30]. Our observation of high ED-B fibronectin expression in bone marrow mesenchymal precursors (MSC and MPC) could contribute to explain this side effect.

MPC can be isolated from bone marrow upon appropriate conditions [31] and thereafter differentiated into blood vessels, bone, cartilage and MSC [32]. Indeed, MSC are cells already committed through differentiation compared to MPC. Interestingly, MSC expressed a much higher degree of ED-B fibronectin that MPC. Therefore, we believe that ED-B fibronectin is a marker of EMT and possibly an interesting target for therapy in more advanced tumors.

## MATERIALS AND METHODS

### Cell culture and TGF-β treatment

Human prostate cancer cell lines PC3 and Du145 were purchased from the American Type Culture Collection (ATCC, Manassas, VA, USA). PC3 and Du145 were cultured respectively in RPMI/F12 1:1 mixture (Life Technologies, Carlsbad, CA, USA) and RPMI 1640 medium (Life Technologies) supplemented with 10% foetal bovine serum (FBS, Sigma-Aldrich, St. Louis, MO, USA), 2mM L-glutamine (Lonza, Wolkersville, MD, USA), 100IU/ml penicillin (Pharmacia & Upjohn S.p.A., Milan, Italy), 100IU/ml streptomycin (Bristol-Myers Squibb S.p.A., Sermoneta, Italy). Cells were cultured at 37°C in 5% CO_2_ incubator. In order to induce EMT, cell lines were treated with 20 ng/ml TGF-β (Miltenyi Biotech, Bergisch Gladbach, Germany) for six passages (2 weeks). Cell detachment has been performed using 0.05%trypsin-0.02%ethylenediaminotetraacetic acid (Life Technologies). The experiments have been performed in triplicate.

### Immunophenotypic analysis

TGF-β untreated and treated cells were analyzed after 72h incubation and at the end of the experiment (6 passageses-2 weeks). The cells were detached and a total of 100 μl of cell suspension (5×10^5^cells) was aliquot per tube. The labelled monoclonal antibodies (mAbs) were incubated for 30 min at 4°C and thereafter samples were washed twice in MACSQuant running buffer (Miltenyi Biotech, Bergisch Gladbach, Germany). The flow cytometer was set using cells stained with isotype controls. Cells were gated on a forward *versus* side scatter plot to eliminate debris. Acquisition was performed using 10,000 cells that were analyzed by MACSQuant cytofluorimeter running the MACSQuantify software (Miltenyi Biotech). Cells were stained using mAbs specific for: EPCAM-APC (Miltenyi Biotech), E-cadherin-PE and N-cadherin-PE (Abcam, Cambridge, UK), CD44-FITC (Miltenyi Biotech), CD105-PE-Vio770 (Miltenyi Biotech), CD90-FITC (Miltenyi Biotech), STRO-1 Alexafluor 647 (Biolegend, San Diego, CA), CD31-PE/Cy7 (Miltenyi Biotech).

### Western blot

Cells were lysed on ice using RIPA Lysis buffer kit with protease inhibitor cocktail (Santa Cruz Biotechnology Inc., Heidelberg, Germany). Protein concentration was measured using a BCA protein assay kit (Pierce, Thermo Scientific, Rockford, U.S.A). Thirty μg of reduced proteins in Laemmli sample buffer (Biorad, Segrate, Milan, Italy) were resolved using Gel precast Miniprotean 4-20% (Biorad) and transferred to nitrocellulose by Semi-Dry Trans-Blot Turbo System (Biorad). Membranes were blocked with 5% non-fat dry milk (Biorad) in TBS-T, and incubated with primary antibody overnight at 4°C and thereafter with the appropriate HRP-conjugated secondary antibody (Biorad) for 1h at room temperature. Membranes were developed using ECL detection reagent (Amersham, Glattbrugg, Switzerland). The primary antibodies were: anti-EP-CAM, anti-E-cadherin, anti-Vimentin (1:1000) and anti-α-Tubulin (1:2000) (Cell Signalling Technology, Massachusetts, USA). Blots were stripped for 20 minutes using the stripping buffer (Thermo Scientific) before re-probing.

### Quantitative RT-PCR

RNA extraction was performed using RNeasy Mini Kit (Qiagen GmbH, Hilden, Germany), according to the manufacturer protocol. One microgram of each RNA sample was retrotranscribed by QuantiTect Reverse Transcription Kit (Qiagen GmbH) and 30-fold dilutions of cDNAs were analyzed by Quantitative RT-PCR on iCycler-iQ5 Optical System (Bio-Rad Laboratories, Hercules, CA), using SsoAdvanced SYBR Green SuperMix (Bio-Rad). All samples were run in duplicate. Primers were designed from coding sequences published on Gene Bank database with the help of Beacon Designer v.7 Software (Premier Biosoft International, Palo Alto, CA, USA); sequences are available upon request. Relative quantitative analysis was performed following 2^−ΔΔCt^ Livak's method [33]. Normalization of PC3 data was performed with *B2M* and *ATP5B*. In Du145 expression analysis, four housekeeping genes (*ACTB, ATP5B, GAPDH, HPRT*) were chosen for normalization on the basis of the GeNorm study.

### Bone marrow-derived multipotent stromal cell cultures

Bone marrow samples were obtained from 10 patients (6M/4F, median age 69 years) undergoing orthopaedic surgery. Mesangiogenic progenitor cells (MPCs) were isolated from bone marrow mononuclear cells (BM-MNCs) cultured in DMEM with 10% pooled human AB serum, obtained from male donors only (PhABS, Lonza, Walkersville MD-USA), as previously described [34]. Mesenchymal stromal cell (MSC) cultures were obtained differentiating MPC in MesenPRO-RS ^®^ medium (Invitrogen, Carlsband CA-USA) for two passages (P2-MSC) [31].
